# Salvianolic Acid B Inhibits Aβ Generation by Modulating BACE1 Activity in SH-SY5Y-APPsw Cells

**DOI:** 10.3390/nu8060333

**Published:** 2016-06-01

**Authors:** Ying Tang, Dan Huang, Mei-Hua Zhang, Wen-Sheng Zhang, Yu-Xin Tang, Zheng-Xiang Shi, Li Deng, Dai-Han Zhou, Xin-Yi Lu

**Affiliations:** 1 The First Affiliated Hospital of Guangzhou University of Chinese Medicine, Guangzhou University of Chinese Medicine, Guangzhou 510006, China; 18825144748@163.com (Y.T.); zdh@oncology.org.cn (D.-H.Z.); 2 Department of Stomatology, Affiliated Hospital of Nantong University, Nantong 226361, China; huangdan114@126.com; 3 Nantong Tumor Hospital, Nantong 226361, China; zhangmeihuaandcyd@163.com; 4 Rugao Changjiang Hospital, Nantong 226532, China; zws238669@163.com; 5 Maternal and Child Health Hospital of Zhoushan, Zhoushan 316000, China; yingying19870722@163.com; 6 Zhoushan Hospital, Zhoushan 316004, China; shizhengxiang507@163.com; 7 Guangzhou Hospital of TCM, Guangzhou 510130, China; denglli@126.com; 8 Biological Resource Center, Second Affiliated Hospital of Guangzhou University of Chinese Medicine, Guangzhou 510120, China

**Keywords:** Alzheimer’s disease, Salvianolic acid B, Aβ generation, BACE1, oxidative stress, GSK3β

## Abstract

Alzheimer’s disease (AD) is a neurodegenerative disease in humans. The accumulation of amyloid-β (Aβ) plays a critical role in the pathogenesis of AD. Previous studies indicated that Salvianolic acid B (SalB) could ameliorate Aβ-induced memory impairment. However, whether SalB could influence the generation of Aβ is unclear. Here, we show that SalB (25, 50, or 100 µM) reduces the generation of Aβ40 and Aβ42 in culture media by decreasing the protein expressions of BACE1 and sAPPβ in SH-SY5Y-APPsw cells. Meanwhile, SalB increases the levels of ADAM10 and sAPPα in the cells. However, SalB has no impact on the protein expressions of APP and PS1. Moreover, SalB attenuates oxidative stress and inhibits the activity of GSK3β, which might be related to the suppression of BACE1 expression and amyloidogenesis. Our study suggests that SalB is a promising therapeutic agent for AD by targeting Aβ generation.

## 1. Introduction

Alzheimer’s disease (AD), an age-related disease, is characterized by progressive neurodegenerative disorders. Senile plaques, loss of neurons, and neurofibrillary tangles are the hallmarks of AD [1]. Amyloid beta (Abeta or Aβ) is the core component of the senile plaques in AD patients’ brains. Aβ triggers subsequent pathological events such as synaptic degeneration, Tau-hyperphosphorylation, oxidative stress, neuroinflammation, neurite degeneration, and neuronal loss, which leads to the progression of AD [2]. Therefore, it is critical to discover novel drugs, targeting Aβ, to improve or halt the progression of the disease. Recently, a number of drug candidates targeting Aβ through immunotherapy have proceeded to clinical trials, but all failed to improve cognitive functions in patients [3]. Traditional Chinese herbal medicine may be one effective method for treating AD. However, further evidence is required before it can be recommended.

**Figure 1:**
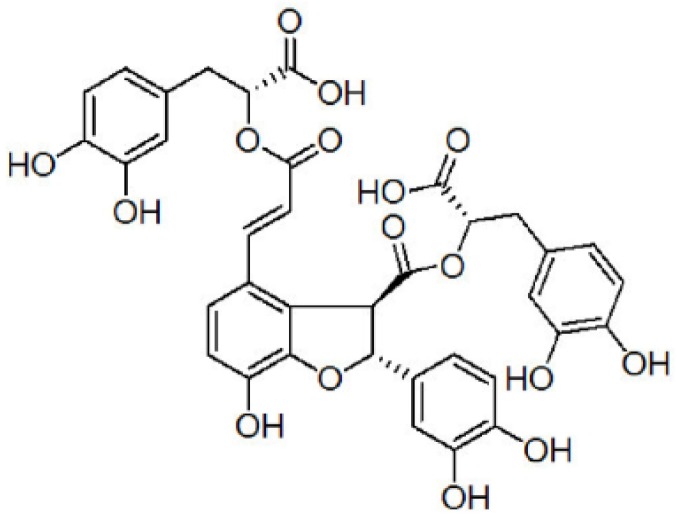
Chemical structure of Salvianolic acid B.

*Salvia miltiorrhiza* is a well-known medicinal plant in the Labiatae family [4]. Because of its remarkable biological activities, *Salvia miltiorrhiza* has also become a widely accepted health-promoting product for the functional food, pharmaceutical, cosmetics, and nutraceutical industries [5]. Salvianolic acid B (SalB; Figure 1) is the most abundant and bioactive compound extracted from *Salvia miltiorrhiza* Bunge [6]. Previous studies have found SalB plays a role in anti-atherosclerosis [7], protects liver injury and reverses liver fibrosis [8]. In addition to its well-established cardioprotective effect, the effect on AD has also been well studied. Lee *et al.* discovered that SalB exerted neuroprotective activity via anti-oxidative and anti-inflammatory actions [9]. Tang *et al.* reported SalB could inhibit Aβ aggregation and fibril formation in PC12 cells [10]. Further, SalB can mediate the GABAergic neurotransmitter system to improve Aβ25-35-induced memory impairment [11]. These results suggest that SalB is a potential therapeutic candidate for AD therapy. However, whether SalB could influence the generation of Aβ is unclear.

Aβ peptides are excised from the amyloid precursor protein (APP), a single-span membrane protein. APP can be cleaved by three proteolytic enzymes: α-, β- and γ-secretase [12]. Under physiological conditions, the majority of APP is cleaved by α-secretase (the extracellular region) into a fragment of 83 amino acids (C83) and an extracellular domain (sAPPα). sAPPα is further cleaved by γ-secretase. The cleavage site of α-secretase can prevent the generation of Aβ. Under pathological conditions, APP is mainly cleaved by β-secretase (the extracellular region) and gives rise to a C-terminal membrane-bound fragment (C99) and an extracellular domain (sAPPβ), which is further cleaved by γ-secretase into Aβ [13]. Thus, inhibition of BACE1 (β-secretase) activity may be an effective way to avoid Aβ accumulation [14]. Aβ is derived from APP in two major forms: 40 amino acid form (Aβ1-40) and more fibrillogenic, the 42 amino acid form (Aβ1-42) [15,16].

We developed an SH-SY5Y cell line overexpressing the human APP Swedish mutant (APPsw) model in this study. In this cell model, we found that SalB pretreatment inhibited β-secretase 1 (BACE1) processing of APP through anti-oxidative stress and regulation of the glycogen synthase kinase 3 beta (GSK3β) signalling pathway.

## 2. Materials and Methods

### 2.1. Drugs, Reagents and Antibodies

SalB (purity > 99%) was purchased from the Chinese National Institute for the Control of Pharmaceutical and Biological Products (Beijing, China). Dulbecco’s modified Eagle’s medium (DMEM), fetal bovine serum (FBS), neurobasal medium, and F12 supplement were obtained from Gibco (New York, NY, USA). 2′,7′-dichlo-rofluorescin diacetate (DCFH-DA) was obtained from Invitrogen (Carlsbad, CA, USA). Assay kits for malondialdehyde (MDA), superoxide dismutase (SOD), and glutathione reductase (GSH-Px) were purchased from Nanjing JianCheng Bioengineering Institute (China). The blots were probed with the following antibodies: anti-APP (Millipore, Boston, MA, USA); anti-sAPPα (Abcam, Cambridge, UK); anti-sAPPβ (Immuno-Biological Laboratories, Fujioka, Japan); anti-BACE1 (Millipore); anti-disintegrin and metalloprotease 10 (ADAM10, Millipore); anti-presenlin 1 (PS1, Millipore), anti-GSK3β (Abcam); anti-pS9-GSK3β (Abcam); anti-β-actin (Sigma-Aldrich, St. Louis, MO, USA); and secondary antibody horseradish peroxidase- (HRP-) conjugated goatanti-rabbit IgG (Cell Signaling Technology, Boston, MA, USA). The Western blot chemiluminescent horseradish peroxidase substrate was purchased from Millipore. All other reagents and chemicals used in the study were of analytical grade.

### 2.2. Cell Culture

SH-SY5Y human neuroblastoma cells transfected with APPsw were cultured in DMEM supplemented with 10% FBS. Cells were kept at 37 °C in a humidified 5% CO_2_/95% air incubator. On the 2nd day after seeding, the medium was changed to serum-free medium 2 h before SalB treatments. Cells were then treated with 25, 50, or 100 µM SalB for 24 h in 6 mL of serum-free culture medium.

### 2.3. ELISA

The cell culture media of SH-SY5Y-APPsw cells were collected. The cell media were centrifuged at 3000 *g* for 5 min to precipitate the cells in the media. The concentration of Aβ40 and Aβ42 were measured by using an ELISA kit (Invitrogen, Carlsbad, CA, USA) according to the manufacturer’s instruction. α- and β-secretase activities were measured by relevant kits according to the manufacturer’s instructions (R&D Systems).

### 2.4. Reactive Oxygen Species (ROS) Production

Intracellular ROS were measured using the redox-sensitive fluorescent dye, DCFH-DA. Conversion of non-fluorescent DCFH-DA to fluorescent dichlorofluorescein (DCF) in the presence of ROS was measured on a microplate reader. In brief, following drug treatment, cells were washed twice with D-Hanks solution, incubated with 10 µM DCFH-DA for 30 min at 37 °C in the dark, and washed twice with D-Hanks solution to remove the extracellular DCFH-DA. Fluorescence emission intensity of DCF (538 nm) was measured in response to 485 nm excitation. The level of intracellular ROS was expressed as a percentage of control cultures incubated in DCFH-DA.

### 2.5. MDA, SOD and GSH-Px Assays

Cells were washed with D-Hanks solution, scraped from the plates into 1 mL ice-cold PBS (0.1 M, containing 0.05 mM EDTA), and homogenized. The homogenate was centrifuged at 4000 *g* for 10 min at 4 °C. The supernatants were stored at −80 °C until analyses. The protein concentration in each supernatant sample was determined using the BCA method. The level of MDA, SOD and GSH-Px activities, and protein content were determined by using specific detection kits according to the manufacture’s instructions. Concentrations were normalized to the sample protein concentration expressed as a percentage of untreated control cultures.

### 2.6. Western Blot Assay

The cells were lysed on ice by precooled lysis buffer. After centrifugation at 12,000 *g* for 15 min at 4 °C, the protein content of the supernatant was determined with a Pierce BCA protein assay kit (Thermo Fisher Scientific, Westminster, MD, USA) to ensure equal sample loading. Protein lysates were separated by SDS-PAGE and blotted onto nitrocellulose membrane (Amersham Biosciences, Piscataway, NJ, USA). Proteins were detected by using antibodies (anti-APP; anti-sAPPα; anti-sAPPβ; anti-ADAM10; anti-BACE1; anti-PS1; anti-GSK3β; anti-pS9-GSK3β; anti-β-actin) and visualized by using anti-mouse or anti-rabbit IgG conjugated with HRP and Pierce ECL Western Blotting Substrate as the substrate of HRP.

### 2.7. Statistical Analysis

Experimental values are presented as the means ± SD. The statistical analysis between two groups was evaluated with Student’s unpaired *t*-test. Statistical analysis of the data among multi-groups was performed using the SPSS 18.0 statistical software (IBM, Armonk, NY, USA). One-way analysis of variance (ANOVA) was applied to analyze differences in the data of the biochemical parameters among the different groups, followed by Dunnett’s significant *post-hoc* test for pairwise multiple comparisons. Differences were considered as statistically significant at *p* < 0.05.

## 3. Results

### 3.1. SalB Reduces the Levels of Aβ40 and Aβ42 in Cell Culture Medium

**Figure 2:**
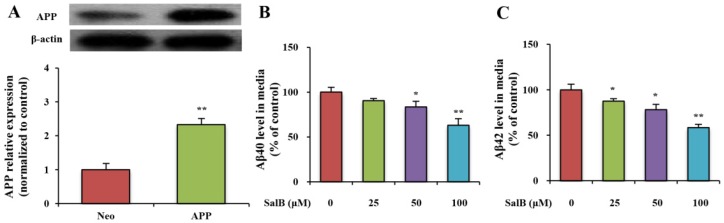
Effect of SalB on Aβ40 and Aβ42 levels in SH-SY5Y-APPsw cells. SH-SY5Y-APPsw cell was developed (**A**); Aβ40 (**B**) and Aβ42 levels (**C**) were measured in SH-SY5Y-APPsw cells. Values are expressed as the mean ± SD in each group (*n* = 3). * *p* < 0.05, ** *p* < 0.01 *vs.* control. SalB: Salvianolic acid B.

To investigate how SalB affects the generation of Aβ, an SH-SY5Y cell line overexpressing the human APP Swedish mutant was developed (Figure 2A). Aβ peptides were produced by cleaving APP. To evaluate the effect of SalB on the viability of SH-SY5Y-APPsw cells, different doses of SalB (25, 50, 100, 200 or 400 µM) were added to the cells. Results showed that 25–200 µM SalB did not affect the viabilities of SH-SY5Y-APPsw cells. However, 400 µM SalB influenced the cell viabilities (Supplemental Material Figure S1A). Thus, the effects of 25–200 µM SalB on the generations of Aβ40 and Aβ42 were further studied by ELISA. SalB (25, 50 or 100 µM) significantly decreased the levels of Aβ40 and Aβ42 in a dose-dependent manner (Figure 2B,C). However, 200 µM SalB was not different from 100 µM SalB, which indicated that 100 µM SalB reached the platform stage (Supplemental Material Figure S1B). In our study, SalB (25, 50 or 100 µM) was employed for further analyses.

### 3.2. SalB Reduces the Level of sAPPβ in SH-SY5Y-APPsw Cells

**Figure 3:**
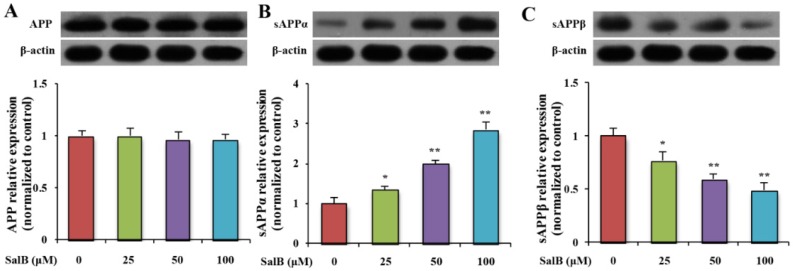
Effect of SalB on the protein expression of APP, sAPPα and sAPPβ in SH-SY5Y-APPsw cells. The protein expressions of APP (**A**); sAPPα (**B**) and sAPPβ (**C**) were determined in SH-SY5Y-APPsw cells. Values are expressed as the mean ± SD in each group (*n* = 3). * *p* < 0.05, ** *p* < 0.01 *vs.* control. SalB: Salvianolic acid B.

To determine whether SalB could affect APP metabolism, the level of full APP was evaluated. Result showed that SalB did not influence the protein expression of total APP (Figure 3A). In addition, the products of α- and β-secretase-mediated APP cleavage (sAPPα and sAPPβ) were examined. SalB dose-dependently increased the level of sAPPα and decreased the level of sAPPβ (Figure 3B,C).

### 3.3. SalB Decreases the Protein Expression and the Activity of BACE1 in SH-SY5Y-APPsw Cells

**Figure 4:**
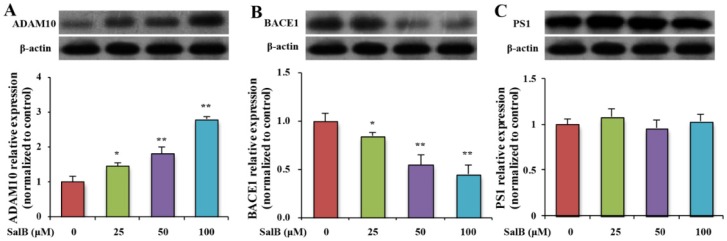
Effect of SalB on the protein expression of ADAM 10, BACE1 and PS1 in SH-SY5Y-APPsw cells. The protein expressions of ADAM 10 (**A**); BACE1 (**B**) and PS1 (**C**) were determined in SH-SY5Y-APPsw cells. Values are expressed as the mean ± SD in each group (*n* = 3). * *p* < 0.05, ** *p* < 0.01 *vs.* control. SalB: Salvianolic acid B.

**Figure 5:**
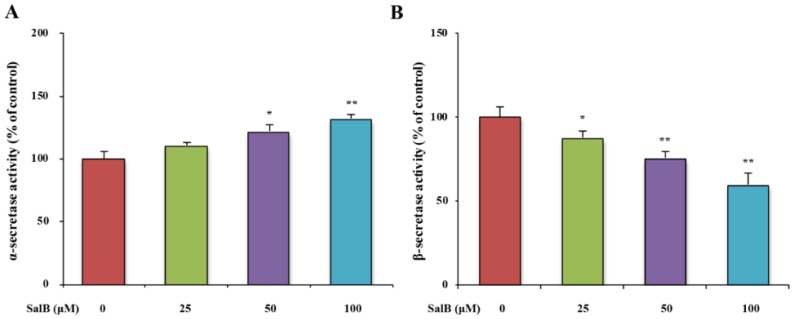
Effect of SalB on α- and β-secretase activities in SH-SY5Y-APPsw cells. α- (**A**) and β-secretase (**B**) activities were measured in SH-SY5Y-APPsw cells. Values are expressed as the mean ± SD in each group (*n* = 3). * *p* < 0.05, ** *p* < 0.01 *vs.* control. SalB: Salvianolic acid B.

In order to analyze whether SalB could affect APP cleavage by modulating APP cleavage enzymes, the protein expressions of ADAM10, BACE1, and PS1 were examined by western blot in SH-SY5Y-APPsw cells. SalB increased ADAM10 expression and decreased BACE1 expression (Figure 4A,B). No statistically significant changes were detected in the protein expression of PS1 (Figure 4C). The activities of α- and β-secretases were further measured by ELISA. Results showed that SalB increased α-secretase activity and decreased β- secretase activity in a dose-dependent manner (Figure 5A,B).

### 3.4. SalB Ameliorates Oxidative Stress and Inhibits GSK3β Activity in SH-SY5Y-APPsw Cells

**Figure 6:**
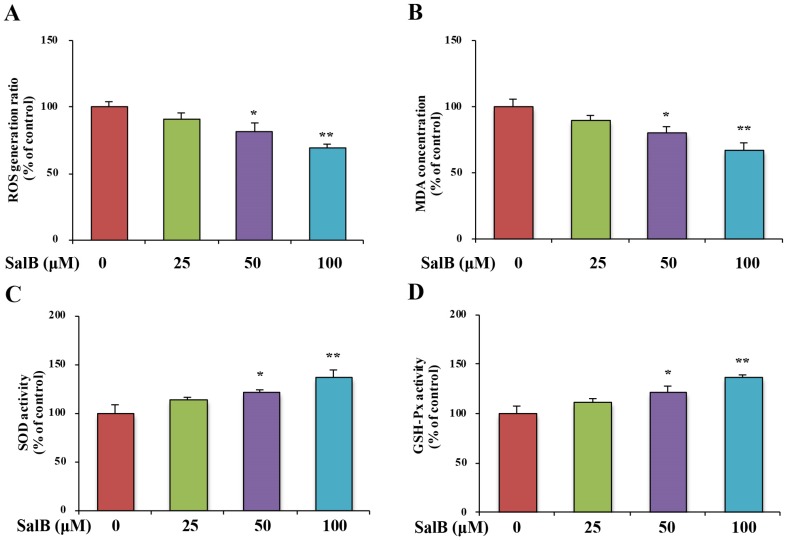

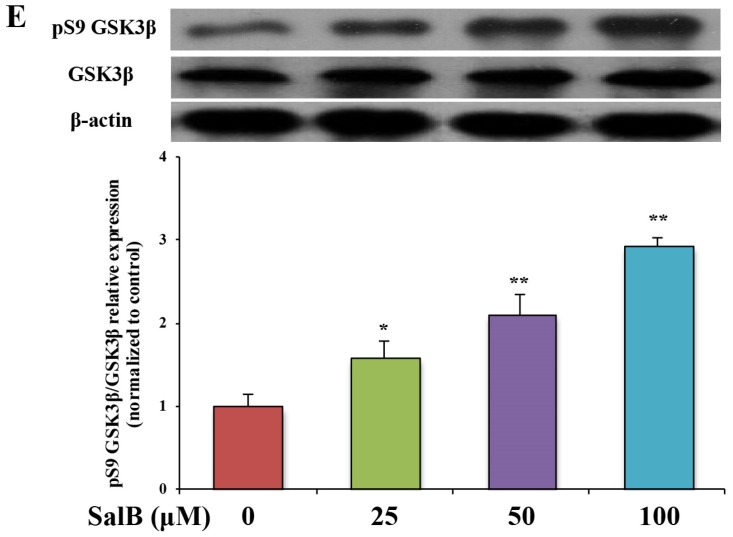
Effect of SalB on oxidative stress and the GSK3β pathway in SH-SY5Y-APPsw cells. ROS level (**A**); MDA level (**B**); SOD (**C**) and GSH-Px (**D**) activities were measured; pS9 GSK3β and GSK3β protein levels (**E**) were determined in SH-SY5Y-APPsw cells. Values are expressed as the mean ± SD in each group (*n* = 3). * *p* < 0.05, ** *p* < 0.01 *vs.* control. SalB: Salvianolic acid B.

Oxidative stress markers in the SH-SY5Y-APPsw cells were examined. After exposure of SH-SY5Y-APPsw cells to SalB for 24 h, intracellular ROS and MDA levels were significantly decreased (Figure 6A,B). In addition, SalB treatment enhanced both SOD and GSH-Px activities in a dose-dependent manner (Figure 6C,D). These results indicate that SalB acts as an antioxidant by directly scavenging free radicals and enhancing endogenous antioxidant capacity. Finally, we found that the phosphorylation at Ser9 of GSK3β, an enzyme well known for its role in the activity of BACE1 [17] and the pathogenesis of AD [18], was significantly increased in SalB-treated group (Figure 6E).

## 4. Discussion

AD is a progressive neurodegenerative disease. Two major factors are known to cause AD, one is extracellular senile plaques and the other is intracellular neurofibrillary tangles in the brain. Aβ is the central component of senile plaques [19]. Finding drugs that target Aβ is an effective intervention for AD treatment. In our study, we found that SalB is a potent drug that can reduce the generation of Aβ in SH-SY5Y-APPsw cells. The suppression of Aβ generation by SalB treatment was associated with alterations of APP processing, including increased α-secretase activity and decreased β-secretase activity. These data demonstrated that SalB inhibits Aβ generation by modulating APP cleavage.

A few studies suggested that SalB, as a crucial neuromodulator, has a direct effect on AD [3,19]. Some studies indicated that SalB can protect Aβ-induced neurotoxicity [9,11]. However, it is unclear whether SalB could have an effect on Aβ generation. Aβ is a product of the cleavage of APP [20]. In the present study, we found that SalB reduced the levels of Aβ40 and Aβ42 in SH-SY5Y-APPsw cells by ELISA. However, SalB did not affect the protein expression of APP. Two major pathways are involved in APP metabolism, one non-amyloidogenic and one amyloidogenic. In the amyloidogenic pathway, APP is first hydrolyzed by BACE1 and generates sAPPβ and CTF-β. γ-secretase further cleaves CTF-β to release AICD and Aβ, which aggregates to form amyloid plaques. In the non-amyloidogenic pathway, APP is cleaved by α-secretase and releases sAPPα and CTF-α. γ-secretase cleaves CTF-α to produce p3 and AICD. ADAM10, located in the Aβ domain, can cleave APP at the α-secretase site, which is involved in the non-amyloidogenic processing of the APP [21,22,23]. Western blot analysis showed SalB increased the protein expressions of ADAM10 and sAPPα and decreased the protein expressions of BACE1 and sAPPβ. However, SalB did not affect the protein expression of PS1. ELISA results demonstrated that SalB increased the activity of α-secretase and decreased the activity of β-secretase. Therefore, we speculated that SalB inhibits the activity of β-secretase, which results in a higher metabolism of APP through the α-secretase pathway and a decrease in Aβ generation.

ROS are by-products of the normal metabolism of oxygen and have an important role in cell signaling [24]. Their concentration is influenced by antioxidant factors. Excessive oxidant conditions result in oxidative stress, which is toxic to cells by damaging proteins, lipids or nucleic acids, ultimately resulting in cell death [25]. Oxidative stress contributes to AD, which has been demonstrated [26,27]. Aβ is a highly redox active peptide that generates ROS [28,29]. ROS promotes several Aβ-driven vicious cycles and propagates the pathogenesis of AD [30]. BACE1 activity is definitely correlated to oxidative stress in AD brains [31]. Treatment of cells with oxidants increases BACE1 transcription, activity and expression [32]. SalB has been reported to exhibit an antioxidant effect [33,34]. Consistently, SalB alleviates the oxidative stress state in SH-SY5Y-APPsw cells. The inhibition of BACE1 may be linked to the antioxidant effect of SalB. In addition, up-regulation of BACE1 is related to the activation of GSK3β [17]. Phosphorylation of certain GSK-3β residues can increase or decrease their ability to bind to the substrate. Phosphorylation at Tyr216 in GSK-3β enhances the enzymatic activity of GSK-3β, while phosphorlyation of Ser9 in GSK-3β significantly decreases active site availability [35]. Results showed SalB could inhibit GSK3β by increasing the ratio of pSer9-GSK3β to total GSK3β. It is likely that SalB suppresses BACE1 expression and amyloidogenesis by attenuation of oxidative stress and the inhibition of the activity of GSK3β. However, the underlying mechanism is unclear and still needs further investigation.

## 5. Conclusions

This study demonstrates that SalB inhibits Aβ generation by modulating APP processing in SH-SY5Y-APPsw cells, and its protective effect is the attenuation of oxidative stress and inhibition of GSK3β signaling. These findings might be considered in the future development of therapeutic strategies for AD.

## Supplementary Materials

The following are available online at http://www.mdpi.com/2072-6643/8/6/333/s1, Figure S1: Effect of SalB on cell viability, Aβ40 and Aβ42 levels in SH-SY5Y-APPsw cells. Cell viability (**A**); Aβ40 (**B**) and Aβ42 levels (**C**) were measured in SH-SY5Y-APPsw cells. Values are expressed as the mean ± SD in each group (*n* = 3). * *p* < 0.05, ** *p* < 0.01 *vs.* control. SalB: Salvianolic acid B.

## References

[1] Qian M.-C., Liu J., Yao J.-S., Wang W.-M., Yang J.-H., Wei L.-L., Shen Y.-D., Chen W. (2015). Caspase-8 mediates amyloid-β-induced apoptosis in differentiated pc12 cells. J. Mol. Neurosci..

[2] Billings L.M., Oddo S., Green K.N., McGaugh J.L., LaFerla F.M. (2005). Intraneuronal aβ causes the onset of early alzheimer’s disease-related cognitive deficits in transgenic mice. Neuron.

[3] Liu Y.-H., Giunta B., Zhou H.-D., Tan J., Wang Y.-J. (2012). Opinion immunotherapy for alzheimer disease-the challenge of adverse effects. Nat. Rev. Neurol..

[4] Zhang Y., Li X., Wang Z. (2013). Diversity evaluation of salvia miltiorrhiza using issr markers. Biochem. Genet..

[5] Wang Y., Peng H., Shen Y., Zhao R., Huang L. (2013). The profiling of bioactive ingredients of differently aged salvia miltiorrhiza roots. Microsc. Res. Tech..

[6] Zhou L.M., Zuo Z., Chow M.S.S. (2005). Danshen: An overview of its chemistry, pharmacology, pharmacokinetics, and clinical use. J. Clin. Pharmacol..

[7] Chen Y.H., Lin S.J., Ku H.H., Shiao M.S., Lin F.Y., Chen J.W., Chen Y.L. (2001). Salvianolic acid b attenuates vcam-1 and icam-1 expression in tnf-alpha-treated human aortic endothelial cells. J. Cell. Biochem..

[8] Lin Y.L., Wu C.H., Luo M.H., Huang Y.J., Wang C.N., Shiao M.S., Huang Y.T. (2006). *In vitro* protective effects of salvianolic acid b on primary hepatocytes and hepatic stellate cells. J. Ethnopharmacol..

[9] Lee Y.W., Kim D.H., Jeon S.J., Park S.J., Kim J.M., Jung J.M., Lee H.E., Bae S.G., Oh H.K., Son K.H.H. (2013). Neuroprotective effects of salvianolic acid b on an a beta(25-35) peptide-induced mouse model of alzheimer’s disease. Eur. J. Pharmacol..

[10] Tang M.K., Zhang J.T. (2001). Salvianolic acid b inhibits fibril formation and neurotoxicity of amyloid beta-protein *in vitro*. Acta Pharmacol. Sin..

[11] Kim D.H., Park S.J., Kim J.M., Jeon S.J., Cho Y.W., Son K.H., Lee H.J., Moon J.H., Cheong J.H., Ko K.H. (2011). Cognitive dysfunctions induced by a cholinergic blockade and a beta(25-35) peptide are attenuated by salvianolic acid b. Neuropharmacology.

[12] Winkler E., Julius A., Steiner H., Langosch D. (2015). Homodimerization protects the amyloid precursor protein c99 fragment from cleavage by gamma-secretase. Biochemistry.

[13] Zhang Y.-W., Xu H. (2007). Molecular and cellular mechanisms for alzheimer’s disease: Understanding app metabolism. Curr. Mol. Med..

[14] Vassar R., Kandalepas P.C. (2011). The beta-secretase enzyme bace1 as a therapeutic target for alzheimer’s disease. Alzheimers Res. Ther..

[15] Thinakaran G., Koo E.H. (2008). Amyloid precursor protein trafficking, processing, and function. J. Biol. Chem..

[16] Lahiri D.K., Farlow M.R., Sambamurti K., Greig N.H., Giacobini E., Schneider L.S. (2003). A critical analysis of new molecular targets and strategies for drug developments in alzheimer’s disease. Curr. Drug Targets.

[17] Ly P.T.T., Wu Y., Zou H., Wang R., Zhou W., Kinoshita A., Zhang M., Yang Y., Cai F., Woodgett J. (2013). Inhibition of gsk3 beta-mediated bace1 expression reduces alzheimer-associated phenotypes. J. Clin. Investig..

[18] Flaherty D.B., Soria J.P., Tomasiewicz H.G., Wood J.G. (2000). Phosphorylation of human tau protein by microtubule-associated kinases: Gsk3 beta and cdk5 are key participants. J. Neurosci. Res..

[19] Kayed R., Head E., Thompson J.L., McIntire T.M., Milton S.C., Cotman C.W., Glabe C.G. (2003). Common structure of soluble amyloid oligomers implies common mechanism of pathogenesis. Science.

[20] Borchelt D.R., Thinakaran G., Eckman C.B., Lee M.K., Davenport F., Ratovitsky T., Prada C.M., Kim G., Seekins S., Yager D. (1996). Familial alzheimer’s disease-linked presenilin 1 variants elevate a beta 1-42/1-40 ratio *in vitro* and *in vivo*. Neuron.

[21] Moir R.D., Tanzi R.E. (2005). Lrp-mediated clearance of abeta is inhibited by kpi-containing isoforms of app. Curr. Alzheimer Res..

[22] Tan J., Mao G., Cui M.-Z., Kang S.-C., Lamb B., Wong B.-S., Sy M.-S., Xu X. (2008). Effects of gamma-secretase cleavage-region mutations on app processing and a beta formation: Interpretation with sequential cleavage and alpha-helical model. J. Neurochem..

[23] Xiao Q., Yan P., Ma X., Liu H., Perez R., Zhu A., Gonzales E., Tripoli D.L., Czerniewski L., Ballabio A. (2015). Neuronal-targeted tfeb accelerates lysosomal degradation of app, reducing a beta generation and amyloid plaque pathogenesis. J. Neurosci..

[24] Devasagayam T.P.A., Tilak J.C., Boloor K.K., Sane K.S., Ghaskadbi S.S., Lele R.D. (2004). Free radicals and antioxidants in human health: Current status and future prospects. J. Assoc. Physicians India.

[25] Valko M., Leibfritz D., Moncol J., Cronin M.T.D., Mazur M., Telser J. (2007). Free radicals and antioxidants in normal physiological functions and human disease. Int. J. Biochem. Cell Biol..

[26] Perry G., Castellani R.J., Smith M.A., Harris P.L.R., Kubat Z., Ghanbari K., Jones P.K., Cordone G., Tabaton M., Wolozin B. (2003). Oxidative damage in the olfactory system in alzheimer’s disease. Acta Neuropathol..

[27] Varadarajan S., Kanski J., Aksenova M., Lauderback C., Butterfield D.A. (2001). Different mechanisms of oxidative stress and neurotoxicity for alzheimer’s a beta(1-42) and a beta(25-35). J. Am. Chem. Soc..

[28] De Felice F.G., Velasco P.T., Lambert M.P., Viola K., Fernandez S.J., Ferreira S.T., Klein W.L. (2007). A beta oligomers induce neuronal oxidative stress through an *N*-methyl-d-aspartate receptor-dependent mechanism that is blocked by the alzheimer drug memantine. J. Biol. Chem..

[29] Huang X.D., Atwood C.S., Hartshorn M.A., Multhaup G., Goldstein L.E., Scarpa R.C., Cuajungco M.P., Gray D.N., Lim J., Moir R.D. (1999). The a beta peptide of alzheimer’s disease directly produces hydrogen peroxide through metal ion reduction. Biochemistry.

[30] Guglielmotto M., Giliberto L., Tamagno E., Tabaton M. (2010). Oxidative stress mediates the pathogenic effect of different alzheimer’s disease risk factors. Front. Aging Neurosci..

[31] Borghi R., Patriarca S., Traverso N., Piccini A., Storace D., Garuti A., Cirmena G., Odetti P., Tabaton M. (2007). The increased activity of bace1 correlates with oxidative stress in alzheimer’s disease. Neurobiol. Aging.

[32] Tong Y., Zhou W., Fung V., Christensen M.A., Qing H., Sun X., Song W. (2005). Oxidative stress potentiates bace1 gene expression and a beta generation. J. Neural Transm..

[33] Wang B., Wang S., Sun J., Shi Y., Le G. (2014). Salvianolic acid b relieves oxidative stress in glucose absorption and utilization of mice fed high-sugar diet. Trop. J. Pharm. Res..

[34] Wu H.-L., Li Y.-H., Lin Y.-H., Wang R., Li Y.-B., Tie L., Song Q.-L., Guo D.-A., Yu H.-M., Li X.-J. (2009). Salvianolic acid b protects human endothelial cells from oxidative stress damage: A possible protective role of glucose-regulated protein 78 induction. Cardiovasc. Res..

[35] Jope R.S., Yuskaitis C.J., Beurel E. (2007). Glycogen synthase kinase-3 (gsk3): Inflammation, diseases, and therapeutics. Neurochem. Res..

