# Supplementary material for: Salvianolic Acid B Inhibits Aβ Generation by Modulating BACE1 Activity in SH-SY5Y-APPsw Cells
Source: Nutrients. 2016 Jun 1;8(6):333. doi: 10.3390/nu8060333 (PMC4924174; doi:10.3390/nu8060333)
Supplement: Supplementary file 1 [file nutrients-08-00333-s001.pdf]

Supplementary Materials: Salvianolic Acid B Inhibits Aβ Generation by Modulating BACE1 Activity in SH-SY5Y-APPsw Cells

Ying Tang, Dan Huang, Mei-Hua Zhang, Wen-Sheng Zhang, Yu-Xin Tang, Zheng-Xiang Shi, Li Deng, Dai-Han Zhou and Xin-Yi Lu

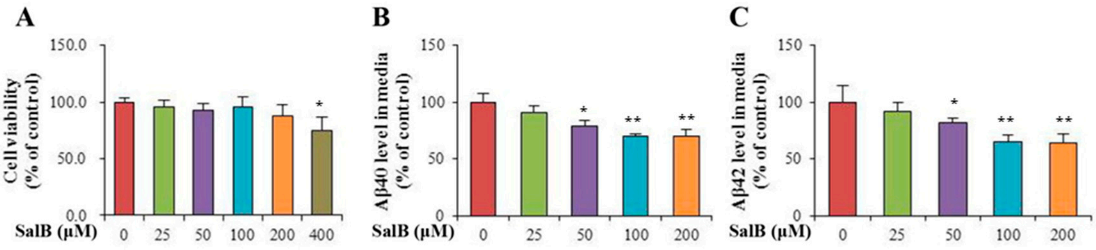

Figure S1. Effect of SalB on cell viability, Aβ40 and Aβ42 levels in SH-SY5Y-APPsw cells. Cell viability (**A**); Aβ40 (**B**) and Aβ42 levels (**C**) were measured in SH-SY5Y-APPsw cells. Values were expressed as mean ± SD in each group ($n = 3$). * $p < 0.05$, ** $p < 0.01$ *vs.* control. SalB: Salvianolic acid B.
